# Yield, growth development and grain characteristics of seven Quinoa (*Chenopodium quinoa* Willd.) genotypes grown in open-field production systems under hot-arid climatic conditions

**DOI:** 10.1038/s41598-023-29039-4

**Published:** 2023-02-03

**Authors:** Mabrouka Oustani, Smail Mehda, Mohammed Tahar Halilat, Haroun Chenchouni

**Affiliations:** 1 Laboratory of Saharan Bio-Resources: Preservation and Development, University of Kasdi Merbah, 30000 Ouargla, Algeria; 2 Department of Agronomy, Faculty of Nature and Life Sciences, University of El Oued, 39000 El Oued, Algeria; 3 Department of Forest Management, Higher National School of Forests, 40000 Khenchela, Algeria; 4 Laboratory of Natural Resources and Management of Sensitive Environments ‘RNAMS’, University of Oum-El-Bouaghi, 04000 Oum-El-Bouaghi, Algeria

**Keywords:** Plant breeding, Plant physiology

## Abstract

Quinoa is an important Andean crop that can play a strategic role in the development of degraded lands in hot arid regions due to its high nutritional value, genetic diversity and its high adaptability to stressful environments. The aim of this work was to evaluate the agronomic performance (growth development, grain yield and grain quality characteristics) of seven quinoa genotypes (Giza1, Sajama, Santa Maria, Q102, Q29, Q27 and Q18) cultivated under open field conditions in the Sahara Desert of Algeria. Using randomized complete block design (4 blocks), field trials were conducted during two cropping seasons (2017–2018 and 2018–2019) from November to April. The measured parameters included: plant height, number of panicles per plant, 1000-grain weight (TGW), grain yield (GYd), grain protein content (GPt), grain saponin content (GSC), and maturity indicators. The genotype effect was statistically the main source of variation in most parameters investigated as compared to the effect of cropping year. The Q102 genotype produced the highest GYd (2.87 t/ha) and GPt (16.7 g/100 g DM); and it required medium period (149 days) to reach harvest maturity as compared to other genotypes. The genotype Giza1 showed the lowest GYd and also low values for most of traits measured. However, it had the shortest harvest maturity (139 days) and the lowest GSC (0.62 g/100 g DM). The variety Santa Maria recorded the highest TGW (2.68 g), but it took 164 days to reach harvest maturity and it had high GSC (1.92 g/100 g DM). Though the best yield and grain quality characteristics were not reunited in single genotype, our findings showed that quinoa has multi-benefit potentials as a new crop for the arid agriculture in particular in hot-arid regions of North Africa.

## Introduction

With the rapid increase in the global population–expected to exceed nine billion by 2050—and the climate change threatening the land and water resources of several regions of the world, the production of enough food for all earth inhabitants is urgently imposed^1,2^. In this context, quinoa is considered as a major complementary crop to counter the food shortages of this century^3,4^. This plant species once was worshiped and called "*chisya mama*" which meant "the mother grain"^5^, is originated from the Andes in South America, mainly from the region of Lake Titicaca between Peru and Bolivia^6,15,64,87,88^.

From a nutritional viewpoint, quinoa has high protein content, with about 14–21% compared to 7–12% for most other cereals (wheat, rice, corn and barley)^3^. Its main nutritional value lies in its balanced and complete composition of essential amino acids—in particular lysine generally lacking in other cereals—comparable to that of milk and superior to that of wheat and other cereals^4,5^. Because of the absence of gluten proteins, quinoa can be used to produce gluten-free cereal-based products, and can thus be eaten by people that have celiac disease and allergy to wheat^7^. Additionally, quinoa contains a high amount of carbohydrates with beneficial hypoglycemic effects while being gluten-free. In addition, it is richer in minerals (Ca, K, P, Mg, Fe and Zn) and vitamins (B1, B9, C and E) than conventional cereals^8,9,81^.

Due to quinoa multi-benefit potentials, several countries started in the last years to promote researches for the development of quinoa cultivation^4,10,11^. This is particularly the case in countries where populations do not have access to sources of protein and/or where food production is limited, and which therefore find themselves obliged to resort to imports or food aid. Quinoa may provide these countries with the ability to meet their food needs on their own. In addition to having a very high nutritional value, quinoa has a huge genetic variability and flexibility, high tolerance to biotic and abiotic stresses, and ability to adapt to adverse soil and climatic conditions in places where most agriculture is marginal^12–14^. Its ability to grow in a wide range of climates and soils, showing a good potential as a grain crop even in new areas outside of its native region, such as cold and subtropical regions in Europe and Asia^15,88,89^ and also the Middle east and North Africa (MENA)^42,55,61,82^. Quinoa tolerance to a wide range of abiotic stresses such as frost, salinity and drought highlight its effectiveness in the fight against desertification^3,6,10,16–19^. According to^20,21^, quinoa not only tolerates salinity, but certain ecotypes of quinoa thrive well in salty soils. For these reasons, the crop is attractive to be cultivated in the hot arid regions. In this context, quinoa offers an excellent alternative crop to ensure food and nutrition security in hot drylands such as the MENA region. Quinoa has a potential use for animal feeding, especially under arid conditions where grain and plant maturity are sometimes difficult to obtain^83^. In fact, the strategy of developing salt-tolerant plants and halophytes as alternative crops to be grown in marginal environments of this region is very important insofar as the major cereal crops are progressively failing to produce satisfied yield under salinity and scarce water resources^21–27^.

Like marginal regions of MENA, in the hot arid land of Algeria, where saline soils occupy large areas and where water is scarce and generally saline^28,29^, utilization of quinoa as halophyte crops would help in highly salinized zones. Consequently, it can generate additional income for local farmers, while contributing to food security and reducing, in the medium and long term, the wheat import bill. The success of trials of the introduction of this culture to the hot-arid conditions of Algeria necessarily requires the study of adaptation and production potential of this plant species under saline conditions, while ensuring that the introduction of this new plant does not harm other crops. On the other hand, despite all its potential of production and adaptation to extreme conditions, the cultivation of quinoa remains limited by many factors that reduce its widespread cultivation at large scale, like sensitivity against temperature, frost, photoperiod, length of cycle duration and the bitter taste due to the presence of saponin which can affect the absorption and digestibility of nutrients^30–32^. The temperature plays an important role for quinoa growth; whereas as hot temperatures results in problems on seeds viability^85^.

The evaluation of new introduced genotypes, resulted from natural crosses among various genotypes is an important target to release new varieties for farmers in drylands of developing countries, such as North African countries. Therefore, it should be possible to select better adapted genotypes with high yields and nutritional quality combined with salt-and drought-tolerance potential. Despite, the several quinoa cultivation trials carried out in hot-arid conditions of the Sahara Desert of Algeria, until now the available published information is very rare regarding quinoa grain yield and quality components under contrasting environments of Algeria. Therefore, the objectives of this study were to (1) evaluate the potential adaptation and production of seven quinoa genotypes under hot-arid conditions of the Sahara Desert, (2) assess the stability of agronomic performance of quinoa genotypes during two consecutive cultivation periods (2017–2018 and 2018–2019), and (3) identify, among superior genotypes for desired traits including: high grain yield, grain weight, low grain saponin content, high grain protein content, optimal growth duration to be used in subsequent genetic improvement programs under hot-arid conditions of the Sahara Desert.

## Materials and methods

### Study area

**Figure 1:**
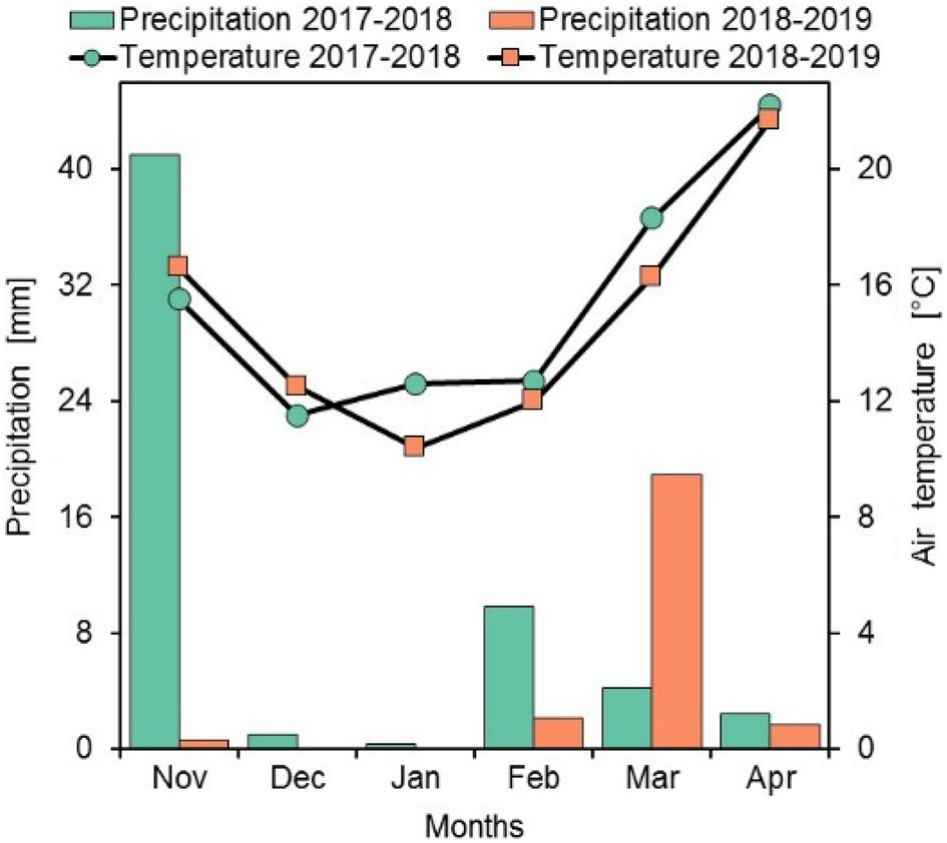
Gaussen ombrothermic diagram for the period November–April of the region of Ouargla (Sahara Desert of Algeria) for two quinoa cropping periods (2017–2018 and 2018–2019).

The trial was conducted in two consecutive growing seasons (2017–2018 and 2018–2019) at a private farm in the commune of Touggourt located in Oued-Righ Valley in Algeria between latitudes from 32°54′ to 39°9′ North and longitudes from 05°50′ to 05°75′ East. Required permission was obtained to collect the plant from the private farm. Please add a statement specifying that permissions or licenses were obtained. The climate of the Touggourt region is hot arid (desertic) with a mean annual precipitation of 54.21 mm of 11 years (2008–2018) where most of the rainfall occurred during October–April. Monthly average of temperature ranged from about 4.8 °C in winter to 43.8 °C in summer^33^. The climatic data recorded during the two experimental growing seasons are shown in Fig. 1. Climatic data were obtained from the weather station of Touggourt^33^. The comparison of averages of monthly temperature and precipitation cumuli during the growing seasons (November to April) from the two years of the experiment showed that mean values of temperatures were very close, with 15.47 and 14.91 °C, respectively. While precipitation in 2017–2018 (58.7 mm) was much lower than in 2018–2019 (23.4 mm). The comparisons of months within experimental periods revealed considerable differences occurred during February and March with 9.8 and 4.2 mm in 2017–2018 compared to 2.1 and 18.9 mm in 2018–2019 (Fig. 1).

The main physical and chemical properties of water and soil (0–60 cm depth) were determined in situ before the beginning of the cultivation trial (Table 1). Pedologically, the soil used in 2017–2018 and 2018–2019 were similar. Their average analytical characterization showed that the soil of study site had sandy texture, alkaline pH (8.46), high electrical conductivity (EC_1/5_ = 4.85 dS/m) and low contents of organic carbon and total nitrogen. The water used for irrigation was characterized by an electrical conductivity of 7.85 dS/m and pH = 8.6. According to the classification established for Algeria by^34^, the water used is inadequate for irrigation in ordinary conditions, but it can be used when soils are permeable with good drainage.

**Table 1:** Physicochemical properties (mean ± standard deviation) of soil (depth 0–60 cm) and irrigation water in study area for two cropping seasons (2017–2018 and 2018–2019).

Soil properties (unit)	Values	Water parameters (unit)	Values
Sand separate (%)	73.67 ± 1.28	pH	8.6 ± 0.5
Silt separate (%)	20.45 ± 1.06	Electrical conductivity (dS/m)	7.85 ± 0.85
Clay separate (%)	5.88 ± 0.8	Sodium adsorption ratio "SAR"	6.11 ± 0.88
pH_(1/2.5)_	8.46 ± 0.47	SO_4_^2–^ (meq/L)	22.45 ± 1.1
Electrical conductivity (dS/m)	4.85 ± 0.65	Cl^–^ (meq/L)	45.23 ± 2.7
Organic matter (%)	0.30 ± 0.06	HCO_3_^–^ (meq/L)	1.59 ± 0.31
Organic carbon (%)	0.17 ± 0.04	CO_3_^2–^ (meq/L)	< 0.01
Total nitrogen (%)	0.029 ± 0.017	SAR class of irrigation water	C_5_–S_1_

### Plant material and experimental design

The study was carried out on seven quinoa (*Chenopodium quinoa* Willd.) varieties provided by the Technical Institute for the Development of Saharan Agronomy (ITDAS of Ouargla and Adrar). These genotypes were selected based on their availability and depending on the variation in their primary origin (Table 2). During the two cropping seasons, the field experiment and trial protocol compared the agronomic performances (precocity indicators and growth, yield, and grain quality of seven quinoa genotypes namely Giza1, Q18, Q29, Q27, Q102, Sajama, and Santa Maria. Sajama, Santa Maria and Q29 was obtained in Bolivia, Q18 and Q29 in Chili, Q102 in Peru, whereas Giza1 was obtained in Egypt (Table 2).

**Table 2:** Some characteristics of the tested quinoa genotypes.

Genotype	Ecotype	Primary origin	Seed color	Seed source
Giza1	Altipolano	Developed in Egypt	Beige	ITDAS of Adrar (Algeria)
Q18	Subtropical	Chili	Yellow	ITDAS of Ouargla (Algeria)
Q27	Sea level	Chili	Light	ITDAS of Ouargla (Algeria)
Q29	Sea level	Bolivia	Yellow	ITDAS of Ouargla (Algeria)
Q102	Salar	Peru	Yellow	ITDAS of Ouargla (Algeria)
Sajama	Not determined*	Bolivia	White	ITDAS of Adrar (Algeria)
Santa Maria	Altipolano	Bolivia	White	ITDAS of Ouargla (Algeria)

*According to^32,69,78^.

The experiment was arranged in a randomized complete block design (RCBD) with four replications for each genotype per block that included 28 elementary plots. The area of each elementary parcel was 28 m^2^ (7 m × 4 m) in which sowing was carried out in ten rows. The spacing between blocks, rows and seedlings was 1.5 m, 0.4 m and 0.35 m, respectively. In order to improve the germination rate, the seeds were refrigerated for a week before sowing, which was carried out manually on 15 November. Sowing was established by dibbling 6–7 seeds per pocket in the soil to a depth of 1–2 cm. Sowing density was 15 kg/ha. To avoid border and interaction effects a buffer area of 1 m preserved between experimental units. Fertilization was applied both before and during the experiment, where 150 kg/ha nitrogen fertilization was divided into two periods, half a dose was applied before sowing as urea, and the rest of nitrogen was applied at flowering stage as Ammonium sulfate (NH_4_SO_4_). Phosphate and potassium fertilizations were applied once using 120 and 100 kg/ha of P_2_O_5_ and K_2_O, respectively. Phosphorus was applied as calcium superphosphate (Ca(H_2_PO_4_)_2_) before sowing, whereas potassium was applied as potassium sulphate (K_2_SO_4_) at the flowering stage. The irrigation supplied every two days and was efficiently applied by drip irrigation. Weeding and phytosanitary treatments were done manually to keep the crop free of weeds throughout the growing season (November–April). Irrigation was stopped a week before harvest which was carried on April. All plots were manually harvested at maturity when the grains became hard, then plants were air-dried for 7 days. The dried panicles for each experimental plot were threshed and winnowed by hand. In this experimental research involving cultivated quinoa plants (no wild variety was used), all methods were performed in accordance with the relevant guidelines and regulations.

### Collection and management of plant data

A composite grain sample of 15 plants was collected randomly from the central rows of each plot (to avoid border effects) during April of both growing seasons, when the grain reached maturity stage (~ 13–14% moisture). These plants and grain samples were the subject of the following measures, that included different kind of variables covering quinoa morphological and physiological growth parameters as well as yield predictors^86^.

#### Morphological growth parameters

***Plant height (PHt):*** the average height from the ground level to the tip of the inflorescence on the main stem was measured at the time of harvesting.

Number of panicles per plant (NPn): was counted at the time of harvest.

#### Precocity indicators

***Number of days to flowering (D-Fl)*:** was recorded as when at least 50% of the flowers were open in all the plants of each plot.

***Number of days to maturity (D-Mt)***: plant cycle duration was taken from date of emergence to the date when the crop was ready for harvesting, i.e. grains had become mature and the plant started drying.

#### Yield variables

***Weight of 1000 grain (TGW):*** a sample of 1000 grains from the bulked grain of each line was weighted (in g).

***Gain yield (GYd)***: was measured, when all plants were dried in the field. The harvested panicles were threshed and grains were extracted and weighted. The grain yield/plot was then converted to tons per hectare (t/ha).

#### Grain quality indicators

The samples of quinoa grains for the seven genotypes were analyzed without shelling, however, grains were visually sorted to remove all impurities. The dried samples were ground to obtain a fine powder for performing the analysis. All chemical analytical determinations were performed in three replicates. Grain samples were dried in an oven at 65 °C until constant weight, and then dried grain were grinded to a fine powder. Afterward, samples were wet digested to be used for determining contents of grain proteins and grain saponins (in g/100 g DM).

***Grain protein (GPt):*** was determined using the Kjeldahl method with a conversion factor of 6.25 following the AOAC method^35^.

***Grain saponin content (GSC)***: was analyzed based on the reverse-phase HPLC procedure described by San Martín and Briones^36^ with slight modifications^37^.

### Statistical analysis

The data collected for morphological growth parameters, precocity indicators, yield variables and grain quality measures were summarized as means and standard deviations for each quinoa genotype tested. Descriptive statistics of these variables for genotypes and cultivation years were represented in boxplots using the package {ggplot2} of the software R that was employed in all statistical tests and graphics^38^. The variation each plant parameter following quinoa genotypes (GT), cultivation years (YR) and the interaction (GT × YR) was tested using two-way analysis of variance (ANOVA) of the RCBD. Then Tukey's post-hoc tests were conducted to distinguish groups of genotypes based on multiple comparisons of means. Pearson correlation tests were applied between all quinoa parameters measured (precocity indicators, growth, yield, grain quality-related parameters) in order to understand the behavior and relationships between plant variables for all genotypes combined under the cultivation conditions in open field of hot-arid lands. The resulting correlation matrix was plotted in an interactive correlation diagram using the package {corrplot} in R. Using the package {nlme} in R, we implanted generalized linear mixed models (GLMMs) with to test the following relationships: effects of PHt, NPn, D-Fl, D-Mt of different genotypes on GYd variation, effect of D-Mt of different genotypes on the variation of PHt, GPt and GSC, and the influence of GYd of genotypes on GPt and GSC. In each model, the block of the RCBD was included as a random effect. In order to overcome model over fitting, so the model only fits the underlying relationships between explanatory variables, we chose the model with the best fit using the backward/forward stepwise selection procedure. Models were compared to each other based on the Bayesian information criterion (BIC), then the model scoring the lowest BIC was selected as the one with the best fit. Statistical significance of all tests was set at *p* < 0.05 and 95% of confidence interval.

## Results

The experimental data were obtained for seven quinoa genotypes, from two growing seasons of replicated field trials, in hot-arid conditions of the Sahara Desert of Algeria. The results summarized in Table 3 indicated that the effect of genotypes was the main source of significant variations. for most parameters investigated as compared to the cropping year and genotype × year interaction effects. Year affected only plant height and precocity parameters (days to flowering and days to harvest maturity). The lack of statistical significance of interaction (genotype × year) for the all traits indicated that the selected genotypes are stable for the characteristics indicated, and therefore the results are processed on the basis of the two-year average.

**Table 3:** ANOVAs for RCBD testing the variation of growth, yield and grain-quality parameters and precocity indicators of quinoa following genotypes and years in the Sahara Desert of Algeria.

Variables	*Df*	*SS*	*MS*	*F*	*p*-value	Sig	*SS*	*MS*	*F*	*p*-value	Sig
		Plant height (PHt)					Number of panicles per plant (NPn)				
Genotype (GT)	6	17,791	2965.1	362.72	< 0.001	***	212.48	35.41	82.39	< 0.001	***
Year (YR)	1	103.0	103.3	12.64	0.001	**	0.93	0.93	2.15	0.150	^ns^
GT × YR	6	76.0	12.6	1.55	0.189	^ns^	1.17	0.20	0.46	0.837	^ns^
Block	3	34.2	11.4				2.40	0.80			
Residuals	39	319.0	8.2				16.76	0.43			

		Days to flowering (D-Fl)					Days to maturity (D-Mt)				
Genotype (GT)	6	726.7	121.11	12.51	< 0.001	***	4947.0	824.6	109.01	< 0.001	***
Year (YR)	1	70.9	70.87	7.32	0.010	*	83.0	82.6	10.92	0.002	**
GT × YR	6	17.3	2.88	0.30	0.935	^ns^	3.0	0.6	0.08	0.998	^ns^
Block	3	73.2	24.40				63.0	21.0			
Residuals	39	377.6	9.68				295.0	7.6			

		Weight of 1000 grains (TGW)					Grain yield (GYd)				
Genotype (GT)	6	5.57	0.93	3.79	0.005	**	8.62	1.44	21.61	< 0.001	***
Year (YR)	1	0.27	0.27	1.09	0.304	^ns^	0.19	0.19	2.92	0.095	^ns^
GT × YR	6	2.02	0.34	1.37	0.250	^ns^	0.14	0.02	0.34	0.913	^ns^
Block	3	0.32	0.11				0.05	0.02			
Residuals	39	9.55	0.24				2.60	0.07			

		Grain protein (GPt)					Grain saponin content (GSC)				
Genotype (GT)	6	125.73	20.96	71.93	< 0.001	***	9.13	1.52	109.76	< 0.001	***
Year (YR)	1	0.18	0.18	0.61	0.439	^ns^	0.01	0.01	0.48	0.493	^ns^
GT × YR	6	2.27	0.38	1.30	0.281	^ns^	0.18	0.03	2.10	0.075	^ns^
Block	3	5.00	1.67				0.04	0.01			
Residuals	39	11.36	0.29				0.54	0.01			

*Df*: degrees of freedom, *SS*: sum squares, *MS*: mean squares, *F*: *F*-statistics, *Sig*.: statistical significance, ***: *p* < 0.001, **: *p* < 0.01, *: *p* < 0.05, ^ns^: *p* > 0.05.

### Morphological growth parameters

#### Plant height

**Figure 2:**
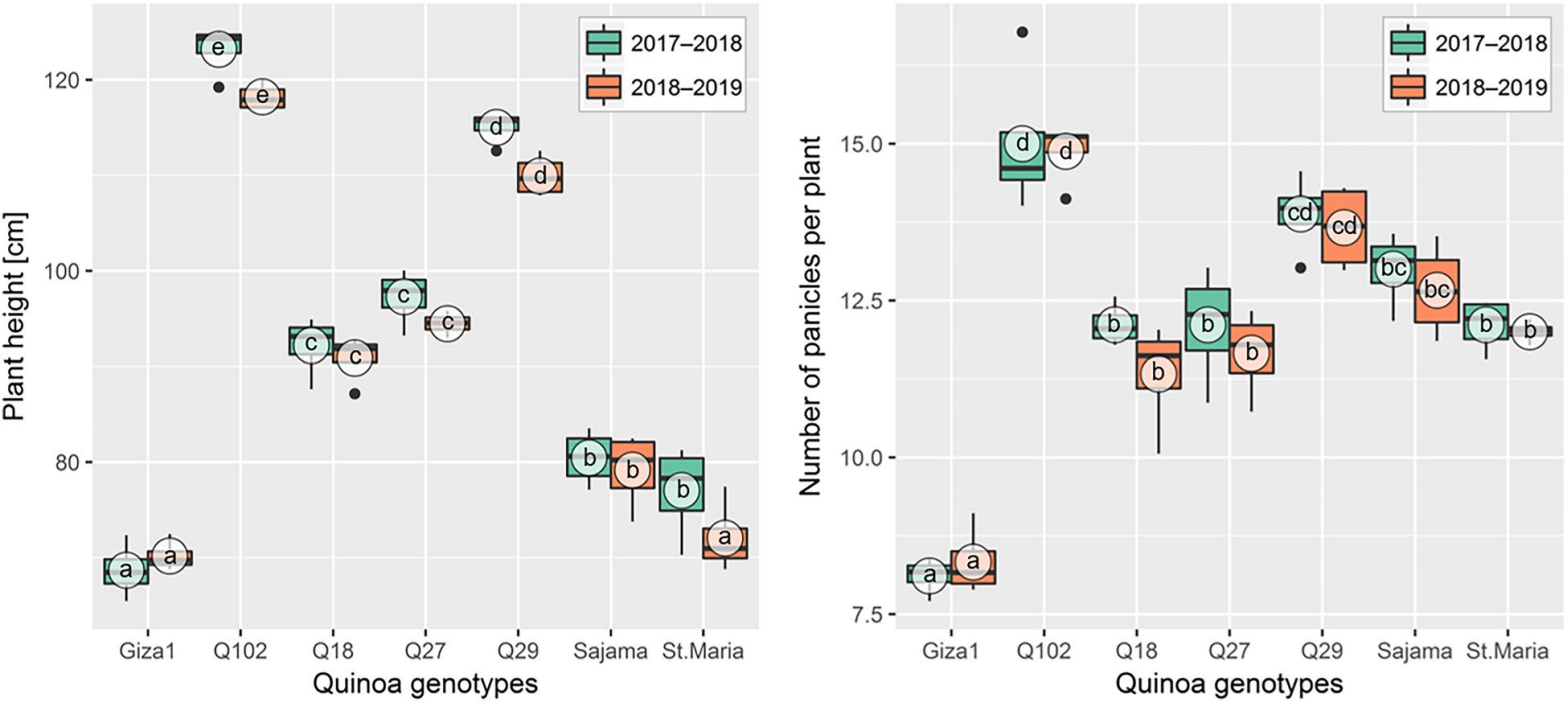
Boxplots showing the variation of plant height and number of panicles during two cultivation seasons for different quinoa (*Chenopodium quinoa*) genotypes grown in open field under hot-arid conditions. The same letters associated with mean values (white circles) are significantly not different (*p* > 0.05) following Tukey's post-hoc test, with data of each cultivation season was tested separately.

The plant height differed significantly (*p* < 0.001) between genotypes and cultivation year (Fig. 2). However, the interaction between these factors was not significant. As regards the two growing seasons, PHt values of the seven genotypes ranged between70.16 cm (Giza1) and 123.2 cm (Q102), with significantly higher values in 2017–2018 for all genotypes combined expect Giza1. The genotype Santa Maria varied the most between the two growing seasons with increment rate of + 7.22% during the first season. While Q18 showed the lowest increment + 1.43% compared to all genotypes tested during the same season. By contrast, Giza1 showed an increase rate of + 2.18% during the second season.

#### Number of panicles per plant

Statistically significant differences (*p* < 0.001) between quinoa genotypes were noted for NPn (Table 3). The highest NPn was recorded in Q102 (14.94 ± 0.87), while the lowest NPn was recorded in Giza1 genotype (8.22 ± 0.42). Compared to other genotypes, Q102 recorded an increment rate of + 81.75, + 27.47, + 25.65, + 23.88, + 16.44 and + 85% for Giza1, Q18, Q27, Santa Maria, Sajama and Q29, respectively (Fig. 2).

### Precocity indicators

**Figure 3:**
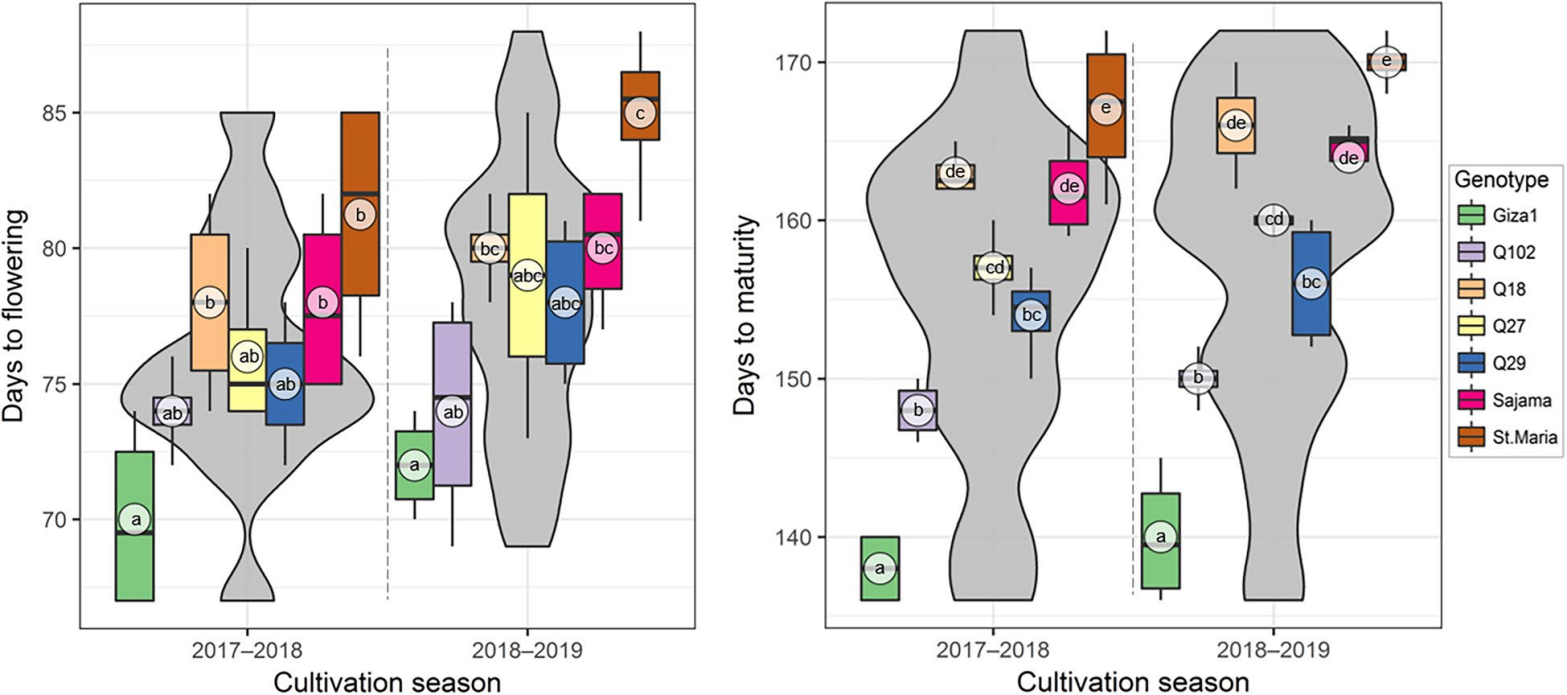
Boxplots overlaid on violin plots showing flowering and maturation periods during two cultivation seasons for different quinoa (*Chenopodium quinoa*) genotypes grown in open field under hot-arid conditions. The same letters associated with mean values (white circles) are significantly not different (*p* > 0.05) following Tukey's post-hoc test, with data of each cultivation season was tested separately.

To estimate the earliness of the genotypes of quinoa tested, two periods was considered, days to flowering and days to maturity. These two periods differed significantly between genotype and year (Table 3). Giza1 presented the shortest time to flowering and to maturity (70 and 138 days), respectively. Santa Maria showed the longest time to flowering and maturity (85 and 170 days), respectively, with significantly higher growth duration for two considered periods in (2018–2019) for all genotypes apart Q102 which recorded the same days to flowering (74 days) for the two growing seasons indicating the stability of this genotype for this period of the growth duration (Fig. 3).Averaged of two years obtained results make it possible to classify the genotypes as regard to their mean total duration growth according to the following decreasing order: Giza1 (139 d) < Q102 (149 d) < Q29 (155 d) < Q27 (158.5 d) < Sajama (163 d) < Q18 (164.5 d) < Santa Maria (168.5 d).

### Yield variables

#### Weight of 1000 grains (TGW)

**Figure 4:**
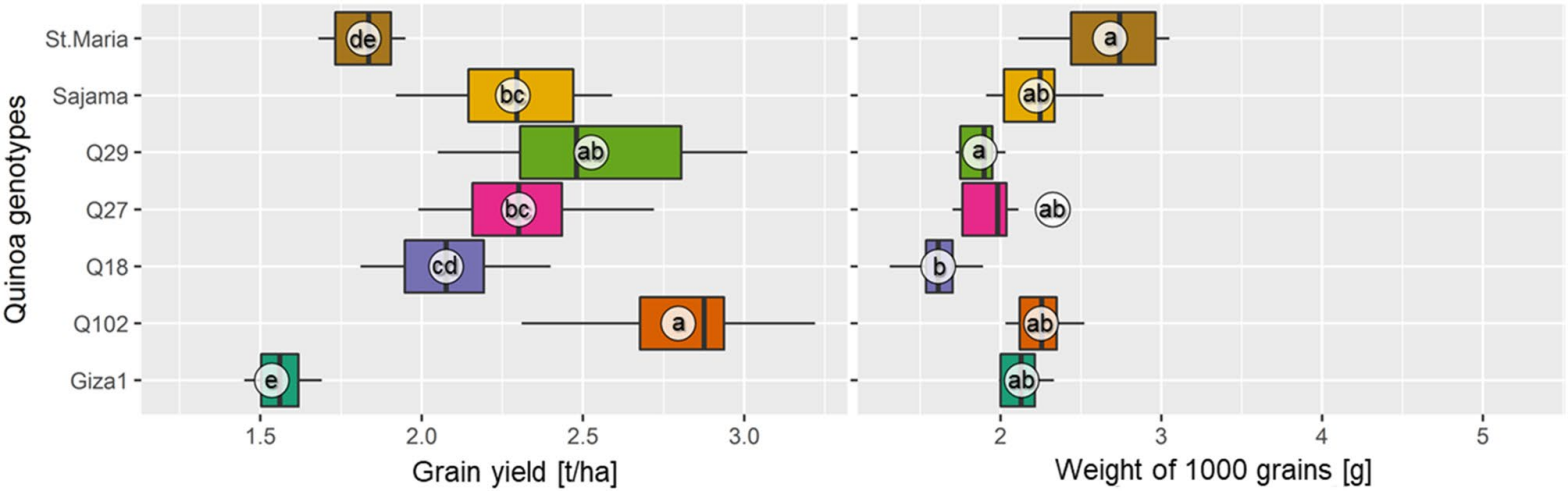
Variation of grain yield and 1000-grain weight for different quinoa (*Chenopodium quinoa*) genotypes grown in open field under hot-arid conditions. The same letters associated with mean values (white circles) are significantly not different (*p* > 0.05) following Tukey's post-hoc test.

The effect of quinoa genotype on weight of 1000 grains was significant (*p* < 0.01) as reported in Table 3. The genotype Santa Maria manifested the highest TGW (2.68 g), however the lowest TGW was recorded in Q18 genotype (1.61 g). The increments in TGW for Santa Maria compared to Q18, Q29, Giza1, Sajama, Q102 and Q27 were + 66.46%, + 43.31%, + 25.82%, + 20.72%, + 19.11% and +15.51%, respectively (Fig. 4).

#### Grain yield

Averaged over (2017–2018) and (2018–2019), Q102 genotype indicted a significantly higher grain yield (2.8 t/ha) compared to the other tested genotypes which recorded the grain yield of 2.52, 2.31, 2.28, 2.07, 1.82 and 1.53 t/ha, respectively for Q29, Q27, Sajama, Q18, Santa Maria and Giza1. In this case, Q102 had an increased GYd by (+ 11.11), (+ 21.21), (+ 22.8), (+ 38.61), (+ 53.84) and (+ 81.81%) in comparison to Q29, Sajama, Q27, Q18, Santa Maria and Giza1, respectively (Fig. 4).

### Grain quality

#### Grain proteins content(GPt)

**Figure 5:**
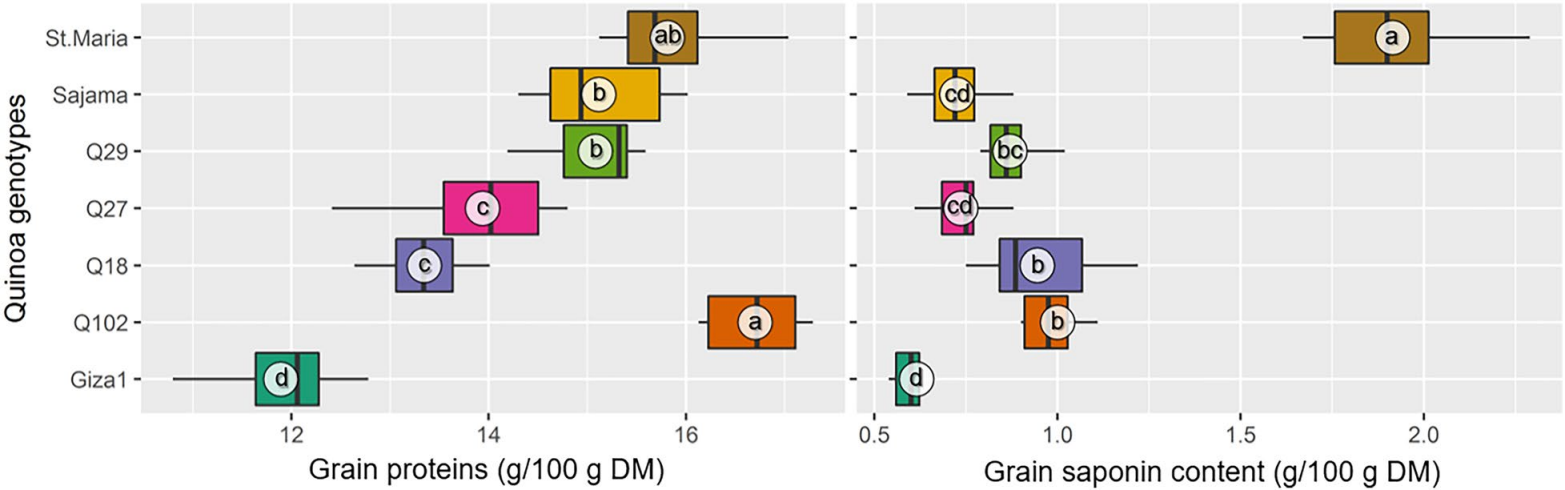
Boxplots displaying protein and saponin contents in the grains of seven quinoa genotypes grown in the Sahara Desert of Algeria. The same letters associated with mean values (white circles) are significantly not different (*p* > 0.05) following Tukey's post-hoc test.

Statistically significant differences were found for GPt in quinoa grains for the seven genotypes tested. Protein content ranged between 11.89 and 16.7 g/100 g DM. The maximum GPt was recorded in Q102 (16.7 ± 0.49 g/100 g DM), while the lowest contend was recorded in Giza1 (11.89 ± 0.68 g/100 g DM) with a reduction rate of − 28.8% of GPt compared to Q102. The other genotypes recorded GPt levels of 15.81 ± 0.62 g/100 g DM, 15.12 ± 0.7 g/100 g DM, 13.94 ± 0.79 g/100 g DM and 13.35 ± 0.46 g/100 g DM for Santa Maria, Sajama, Q29, Q27 and Q18, respectively (Fig. 5).

#### Grain saponin content (GSC)

Statistically significant differences between quinoa genotypes were noted for GSC (*p* < 0.001) (Table 3). Regarding the average values of the two growing seasons, Santa Maria genotype showed a significantly higher GSC (1.92 g/100 g DM) compared to the other genotypes. In comparison to Santa Maria, the recorded GSC in Giza1 (0.62 g/100 g DM), Sajama and Q27 that presented similar two-year averages around 0.73 g/100 g DM, Q27 (0.87 g/100 g DM), Q18 (0.95 g/100 g DM) and Q102 (1 g/100 g DM) were lower by 67.7%, 62%, 54.68%, 50.52% and 48%, respectively (Fig. 5).

### Interrelations between plant parameters

**Figure 6:**
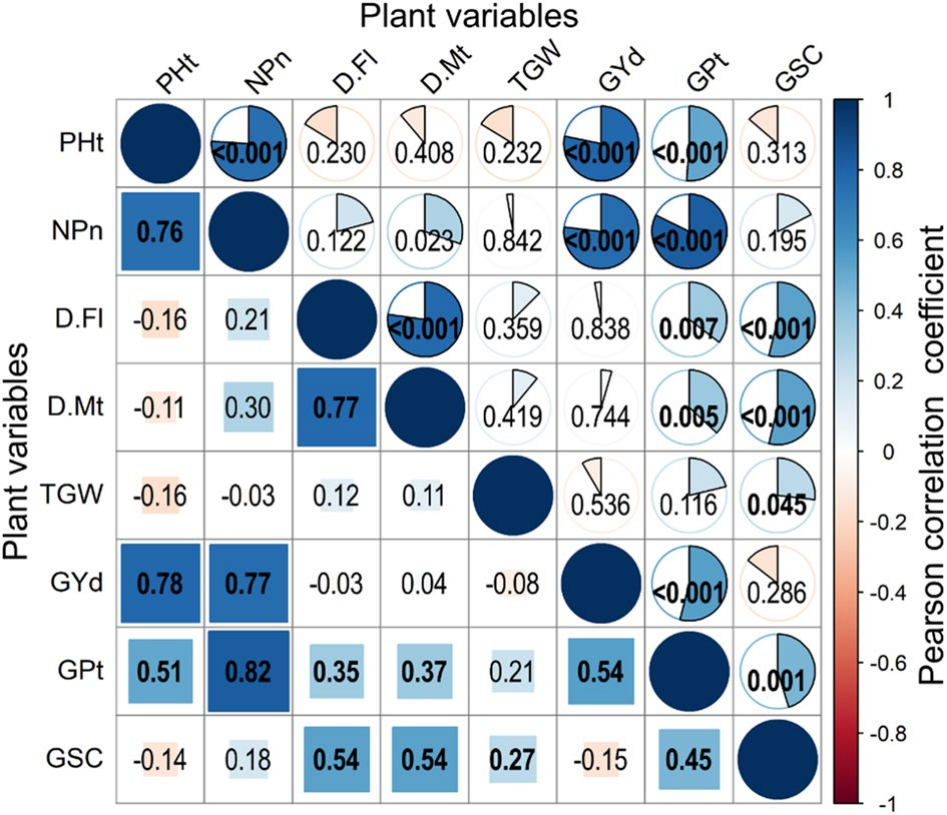
Correlation matrix applied between yield, growth development parameters and grain quality characteristics of quinoa (*Chenopodium quinoa*) genotypes grown in the Sahara Desert. Pearson correlation tests are given as correlation coefficients (values below the diagonal, color shading, pie chart and square sizes) and the *p*-value (values above the diagonal). Significant correlations (*p* ≤ 0.05) are displayed in boldface text.

Pearson correlation coefficients—and associated *p*-values—between various traits are presented in Fig. 6. Correlation tests indicated that relationships between morphological growth parameters were significantly and positively correlated amongst themselves and with (GYd and GPt). Correlations were particularly strong between PHt—NPn (*p* < 0.001), GYd—PHt (*p* < 0.001), GYd—NPn (*p* < 0.001), PHt—GPt (*p* < 0.001) and NPn—GPt (*p* < 0.001). The relationships between yield parameters and grain quality parameters revealed also a strong positive correlation between GYd—GPt (*p* < 0.001). No significant correlation (*p* > 0.05) was found between GYd in relation to TGW, D-Fl and D-Mt. However, the two parameters of precocity were positively correlated amongst themselves (*p* < 0.001) and with GSC (*p* < 0.001).

### Variables influencing grain yield for different genotypes

**Figure 7:**
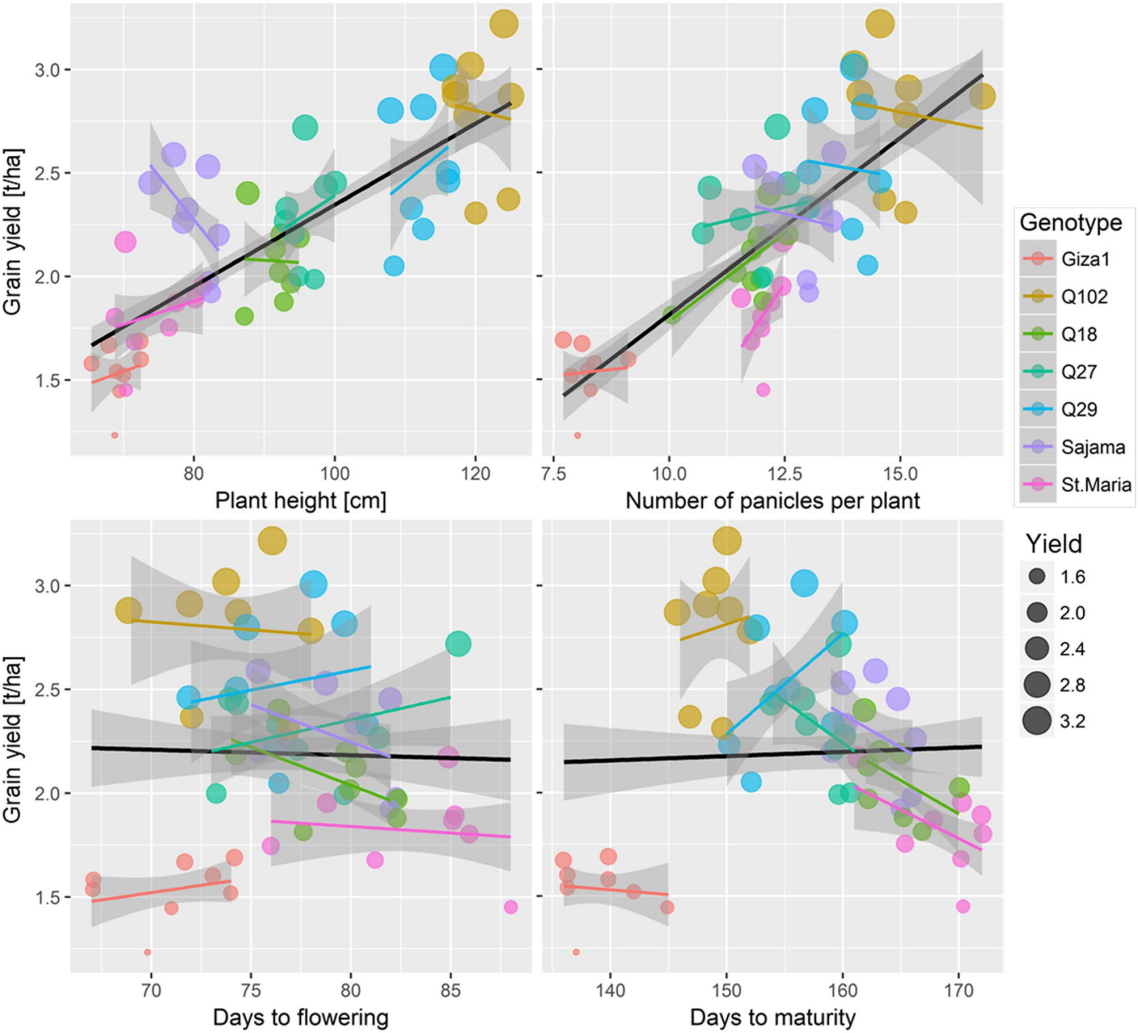
Relationship between grain yield, plant height, number of panicles and maturity duration for different quinoa (*Chenopodium quinoa*) genotypes grown in open field under hot-arid conditions. The color of points is mapped to genotypes, and size values are set to grain yield. The solid lines (black for all genotypes combined, colors for studied genotypes) represent a linear regression with a GLM fit, with 95% confidence regions in light gray.

The GLMM with the best fit (AIC = 55.9) selected from the model testing the effects of PHt, NPn, D-Fl, D.Mt in interaction with quinoa genotypes on the variation of GYd (AIC = 144.63), indicated that the significant predictors of GYd included PHt, NPn, D-Mt and the interaction "PHt × NPn" (Table4). Grain yield seemed to significantly increase with the increase in PHt (*t* = 2.874, *p* = 0.006) and NPn (*t* = 2.967, *p* = 0.005), while D-Mt (*t* = − 2.158, *p* = 0.036) and PHt × NPn (*t* = − 2.355, *p* = 0.023) had significant negative effects on GYd changes. The effects of these variables on GYd differed from on genotype to another (Fig. 7).

**Table 4:** Parameters of generalized linear mixed-effects models (Gaussian fit and identity link) testing the effects of plant height (PHt), number of panicles (NPn), period from emergence to maturity (D-Mt) on the variation of grain yield of quinoa grown under field conditions of the Sahara Desert.

Variables	Estimate	LLCI	ULCI	Std. Error	*DF*	*t*-value	*p*-value
(Intercept)	− 1.785	− 3.996	0.426	1.100	48	− 1.623	0.111
PHt	0.056	0.017	0.095	0.019	48	2.874	0.006
NPn	0.452	0.146	0.758	0.152	48	2.967	0.005
D-Mt	− 0.015	− 0.030	− 0.001	0.007	48	− 2.158	0.036
PHt × NPn	− 0.004	− 0.007	− 0.001	0.002	48	− 2.355	0.023

*DF*: degrees of freedom, LLCI and ULCI: lower and upper limit of the confidence interval, respectively.

### Maturation of vegetative growth and grain quality variables for different genotypes

**Figure 8:**
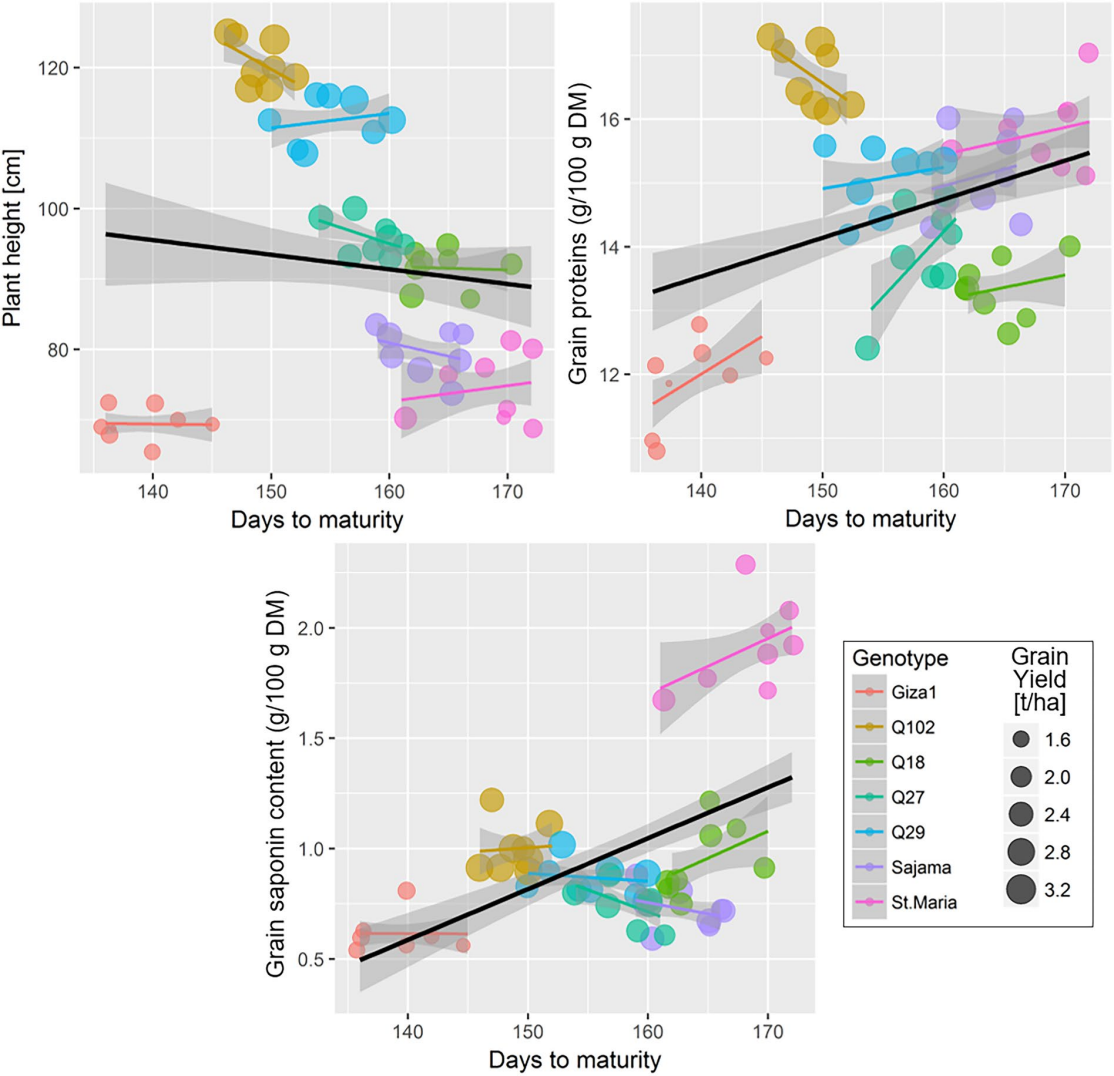
Relationship between maturity duration, plant height, and grain nutritional quality for different quinoa (*Chenopodium quinoa*) genotypes grown in open field under hot-arid conditions. The color of points is mapped to genotypes, and size values are set to grain yield. The solid lines (black for all genotypes combined, colors for studied genotypes) represent a linear regression with a GLM fit, with 95% confidence regions in light gray.

Table 5 summaries the GLMMs testing the variation of PHt, GPt and GSC following the effects D-Mt for different quinoa genotypes. The overall effect of D-Mt on PHt for all quinoa genotypes combined was not significant (*p* = 0.221), however this effect varied significantly among genotypes (*F* = 260,* p* < 0.001). The GLMM revealed that PHt increased significantly (*p* < 0.05) with the increase in D-Mt in every genotype when compared to Giza1 (taken as the intercept in the model). The duration of vegetative maturation did not significantly influence the changes in GPt (*p* = 0.406) and GSC (*p* = 0.539). But, some quinoa genotypes showed significant increases in GPt and GSC with the maturation of vegetative growth. This concerned the maturation of genotype Q102, Q27, Q29, Sajama and St. Maria for GPt; and the genotypes Q102 and St. Maria for GSC (Table 5, Fig. 8). It is noteworthy mentioning that GLMMs examining the effect of D-Mt without including the genotype indicated significant positive relationships (Fig. 8) for the GPt (*F*_(1,51)_ = 0.69,* p* = 0.401) and GSC (*F*_(1,51)_ = 8.55,* p* = 0.005).

**Table 5:** Parameters of generalized linear mixed-effects models testing the variation of plant height (PHt), grain proteins (GPt) and grain saponin content (GSC) following the plant maturation (D-Mt) of seven quinoa genotypes cultivated in open field under hot-arid conditions of the Sahara Desert.

Parameters	Value	LLCI	ULCI	*DF*	*SE*	*t*-value	*p*-value
GLMM: PHt ~ (D-Mt + D-Mt × Genotype)							
(Intercept)	100.532	50.082	150.983	25.048	45	4.014	< 0.001
D-Mt	− 0.224	− 0.587	0.139	0.180	45	− 1.241	0.221
D-Mt × Genotype[Q102]	0.359	0.325	0.393	0.017	45	21.427	< 0.001
D-Mt × Genotype[Q18]	0.169	0.109	0.230	0.030	45	5.630	< 0.001
D-Mt × Genotype[Q27]	0.194	0.144	0.244	0.025	45	7.814	< 0.001
D-Mt × Genotype[Q29]	0.301	0.257	0.345	0.022	45	13.783	< 0.001
D-Mt × Genotype[Sajama]	0.097	0.039	0.154	0.029	45	3.357	0.002
D-Mt × Genotype[St. Maria]	0.070	0.002	0.137	0.033	45	2.082	0.043
GLMM: GPt ~ (D-Mt + D.Mt × Genotype)							
(Intercept)	8.215	− 0.631	17.061	4.392	45	1.871	0.068
D-Mt	0.026	− 0.037	0.090	0.032	45	0.838	0.406
D-Mt × Genotype[Q102]	0.030	0.025	0.036	0.003	45	10.589	< 0.001
D-Mt × Genotype[Q18]	0.005	− 0.006	0.015	0.005	45	0.898	0.374
D-Mt × Genotype[Q27]	0.010	0.001	0.018	0.004	45	2.231	0.031
D-Mt × Genotype[Q29]	0.018	0.010	0.025	0.004	45	4.705	< 0.001
D-Mt × Genotype[Sajama]	0.016	0.006	0.026	0.005	45	3.162	0.003
D-Mt × Genotype[St. Maria]	0.019	0.007	0.030	0.006	45	3.188	0.003
GLMM: GSC ~ (D-Mt + D-Mt × Genotype)							
(Intercept)	0.044	− 1.814	1.902	0.922	45	0.048	0.962
D-Mt	0.004	− 0.009	0.017	0.007	45	0.618	0.539
D-Mt × Genotype[Q102]	0.002	0.001	0.004	0.001	45	3.779	< 0.001
D-Mt × Genotype[Q18]	0.001	− 0.001	0.004	0.001	45	1.248	0.219
D-Mt × Genotype[Q27]	0.000	− 0.002	0.002	0.001	45	0.335	0.739
D-Mt × Genotype[Q29]	0.001	0.000	0.003	0.001	45	1.527	0.134
D-Mt × Genotype[Sajama]	0.000	− 0.002	0.002	0.001	45	0.066	0.948
D-Mt × Genotype[St. Maria]	0.007	0.005	0.009	0.001	45	5.708	< 0.001

*DF*: degrees of freedom, LLCI and ULCI: lower and upper limit of the confidence interval, respectively.

### Relationship between grain yield and grain quality of different genotypes

**Figure 9:**
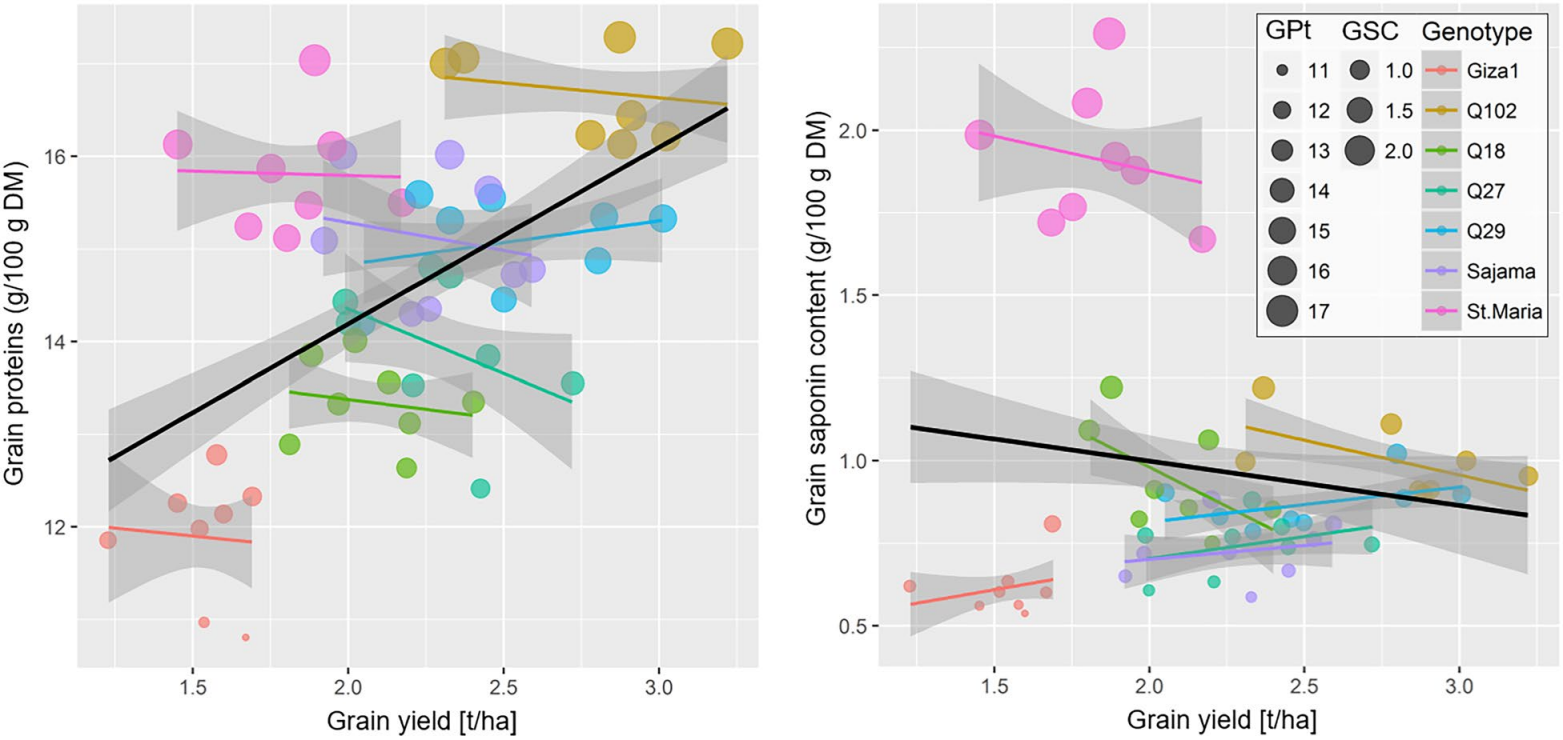
Relationship between grain yield and grain nutritional quality for different quinoa (*Chenopodium quinoa*) genotypes grown in open field under hot-arid conditions. The color of points is mapped to genotypes, and size values are set to values of grain proteins (GPt) and saponins (GSC). The solid lines (black for all genotypes combined, colors for studied genotypes) represent a linear regression with a GLM fit, with 95% confidence regions in light gray.

The analysis of the relationships GYd—GPt and GYd—GSC without considering the genotype as fixed effect in the GLMM revealed highly significant effect of GYd on GPt (*F*_(1,51)_ = 22.33, *p* < 0.001) and a non-significant relation for GSC (*F*_(1,51)_ = 1.16, *p* = 0.287). When including the effect of genotypes alongside the GYd, the latter seemed to have no significant influence on the variation of both GPt and GSC (Table 6). The linear trend of these relationships for each genotype showed curves with slight slopes (Fig. 9).

**Table 6:** Parameters of GLMMs testing the effect of grain yield (GYd) on grain proteins (GPt) and grain saponin content (GSC) for different quinoa genotypes cultivated in open field under hot-arid conditions.

Parameters	Value	LLCI	ULCI	*DF*	*SE*	*t*-value	*p*-value
GLMM: GPt ~ (GYd × Genotype)							
(Intercept)	12.412	7.910	16.914	39	2.226	5.577	< 0.001
GYd	− 0.340	− 3.252	2.572	39	1.440	− 0.236	0.815
Genotype[Q102]	5.443	− 0.412	11.299	39	2.895	1.880	0.068
Genotype[Q18]	2.113	− 4.271	8.497	39	3.156	0.670	0.507
Genotype[Q27]	6.131	− 0.165	12.427	39	3.113	1.970	0.056
Genotype[Q29]	2.792	− 2.771	8.355	39	2.750	1.015	0.316
Genotype[Sajama]	3.408	− 2.590	9.406	39	2.965	1.149	0.257
Genotype[St. Maria]	2.280	− 3.576	8.136	39	2.895	0.787	0.436
GYd × Genotype[Q102]	− 0.073	− 3.268	3.122	39	1.580	− 0.046	0.963
GYd × Genotype[Q18]	− 0.229	− 3.864	3.407	39	1.797	− 0.127	0.899
GYd × Genotype[Q27]	− 1.663	− 5.169	1.843	39	1.733	− 0.960	0.343
GYd × Genotype[Q29]	0.291	− 2.886	3.468	39	1.571	0.185	0.854
GYd × Genotype[Sajama]	0.031	− 3.361	3.423	39	1.677	0.019	0.985
GYd × Genotype[St. Maria]	0.954	− 2.610	4.519	39	1.762	0.542	0.591
GLMM: GSC ~ (GYd × Genotype)							
(Intercept)	0.350	− 0.642	1.342	39	0.490	0.714	0.480
GYd	0.173	− 0.471	0.816	39	0.318	0.543	0.591
Genotype[Q102]	1.225	− 0.079	2.528	39	0.644	1.901	0.065
Genotype[Q18]	1.573	0.156	2.990	39	0.701	2.246	0.031
Genotype[Q27]	0.079	− 1.270	1.429	39	0.667	0.119	0.906
Genotype[Q29]	0.216	− 1.014	1.446	39	0.608	0.355	0.725
Genotype[Sajama]	0.199	− 1.128	1.527	39	0.656	0.304	0.763
Genotype[St. Maria]	1.977	0.689	3.264	39	0.637	3.104	0.004
GYd × Genotype[Q102]	− 0.378	− 1.088	0.332	39	0.351	− 1.078	0.288
GYd × Genotype[Q18]	− 0.644	− 1.450	0.162	39	0.399	− 1.616	0.114
GYd × Genotype[Q27]	− 0.036	− 0.793	0.721	39	0.374	− 0.096	0.924
GYd × Genotype[Q29]	− 0.052	− 0.756	0.652	39	0.348	− 0.150	0.882
GYd × Genotype[Sajama]	− 0.096	− 0.845	0.654	39	0.371	− 0.258	0.798
GYd × Genotype[St. Maria]	− 0.399	− 1.183	0.386	39	0.388	− 1.028	0.310

*DF*: degrees of freedom, LLCI and ULCI: lower and upper limit of the confidence interval, respectively.

## Discussion

This study showed that quinoa has good adaptation and can be successfully cultivated in marginal areas of hot-arid lands of Algeria. The crop was successful during the season November–April. Indeed, quinoa cultivation depend on the adaptation of genotypes to climate and soil conditions. The non-variation of the most studied parameters during both years can be confirmed as there were no annual significant differences, and consequently can be proven that quinoa is generally suitable for cultivation under hot-arid conditions of the Sahara Desert. In this experiment quinoa was cultivated under irrigation. The crop is also established in some countries of the MENA region^42,55,61,82^. In fact, the stability of most parameters from one year to another facilitates the program of breeding for identifying a suitable genotype to grow in this region. On the other hand, the genotypic variability was well demonstrated in our study (most parameters showed significant variations). It should be noted that the conditions of the field trial allowed each genotype to express its potential separately. While, some genotypes are shown to be interesting in terms of yield parameters, growth duration others have shown high potential in terms of quality (GPt and GSC).

### Morphological growth parameters

The plant height of quinoa varied significantly between genotypes (Table 3). These differences may have resulted from genetic structures variability between them. According to^39–41^, plant heights were different in quinoa varieties and populations. Our results are lower than those indicated by Shams^42^ where PHt varied between 135 and 146 cm under almost similar agronomic conditions (sowing in November and in sandy soil) which can be probably explained by differences in other crop practices in particular nitrogen fertilizer (214.2 kg N/ha) compared to our case (150 kg N/ha). According to Geren^43^, quinoa PHt increased noticeably by increasing nitrogen fertilizer rate. This agricultural practice leads to the stimulation of metabolic activity which contribute to the increase in amount of metabolites and consequently lead to internodes elongation and increase plant height^44–46^. However, our results are relatively higher compared to those reported by^47^, which recorded plant heights ranged between 20.3 and 108.3 cm/plant, which may be due to lower temperature. According to^48^, low temperatures slowdown plant growth due to the decrease in enzyme activity in these conditions. Moreover, our results are not in agreement with^49^, which reported that PHt was related to the duration of maturity and generally shorter varieties showed earlier characteristics. In our case genotype with late and medium maturity, like Q102 and 29 grew taller than genotype with early maturity as Giza1 (Fig. 8).

In the present study, the PHt variability illustrated by the significant year effects was probably due to the different weather conditions during the two growing seasons. Plant height was highly variable between the two growing seasons, with the higher values in 2017–2018 (when it was wetter 58.7 mm, Fig. 1). The yearly variability of PHt due to different weather conditions, as it was signaled in the present study, has been highlighted in previous studies^50,51^.

The number of panicles in the present study is low compared to the number reported by^27^, which indicated mean values ranging between 13.44 and 16.16 panicle/plant. Our results are close to those recorded by^52^ (NPn = 14.6–15.8 panicles/plant). The genotypic variability of NPn has been previously reported for quinoa plant^21,42^, with the genotype Q102 recording the highest NPn. The combination of taller PHt, larger NPn and longer growth cycle may be the reason that Q102 and Q29 genotypes exhibited the greatest grain yield among the tested genotypes. Our results showed strong positive correlations between the morphological parameters and grain yield.

### Precocity indicators

Earliness is the most important factor to consider for introducing the quinoa into a new environment and or region. It corresponds to the duration of vegetation required to reach harvest maturity. The length of the quinoa vegetative cycle depends on the physiological state of the seeds planted, on all the agro-climatic factors, cultivation practices and on the genotypes used^53^. The total growth period under conditions of this study is shorter than that reported in South America (110–190 days)^54^, but similar to that reported in Northern India (109–163 days)^15^ and in South of Egypt (115–160 days)^42^. Hirich et al.^24^ under similar sandy soil conditions and sowing date recorded 121 days in South of Morocco. In Dubai, the harvest maturity varied from 93 to 122 days^22^*.* While in Egypt, in Sharqiya Governorate^55^ and in Ismailia Governorate^42^ a range of 115–160 days and 135.5–139 days was recorded, respectively. The great genetic variability observed between the seven genotypes for harvest maturity in the current study is due to differences in regional ecological conditions of the seed origin including soil, rain temperature and altitude^42^. In fact, environmental conditions of southern Algeria have greatly influenced the growth duration of the studied genotypes of quinoa, which is predominantly a self-pollinated species. This character justifies the strong variation reported between genotypes for the cycle duration^26^.

The total growth period of quinoa at the experimental site is quite long for certain genotypes such as Q29 (155 days), Q27 (158.5 days), Sajama (163 days), Q18 (164.5 days) and Santa Maria (168.5 days) in comparison to the generally recommended days growing period of quinoa (< 150 day)^53^. Therefore, these genotypes can be considered as long-duration genotypes under our conditions. Meanwhile Giza1 required only 138–140 days to reach maturity, and thus can be considered as short-duration genotype. Concerning Q102 was occupied an intermediate position between the two groups and therefore this genotype can be considered as medium in its maturity duration (149 days) in the first season and 150 days in the second one. According to^53^, Giza1 and Q102 are considered as beneficial because their growth periods are shorter than 150 days.

It seems that arid climatic and edaphic conditions of hot arid lands of Algeria had a major role in growth duration of the studied quinoa genotypes. The variation in the duration of harvest maturity among genotypes may be explained by their genetic response to low temperature during the growing period. Hirich et al.^24^ showed that low temperatures negatively affected the productivity of quinoa by increasing the duration of the growing period. According to^56^, the optimal temperature for growing quinoa is approximately 22 °C. However, for both cultivation seasons of this study, the temperature at the time of vegetative growth was lower at the optimum growing temperature (Table 3).

The observed difference in D-Mt between the two cropping seasons of this study is probably ascribed to the different weather conditions between years. Indeed, 2018–2019 was drier than 2017–2018 (Fig. 1), which probably caused the shorter crop cycle during the first cropping season. Moreover, the rainfall recorded in the first growing season (2017–2018) was concentrated in November at the beginning of the crop cycle (rainfall = 58.7 mm), and so it provided high levels of air humidity and soil moisture which played a major role in maturity stage of quinoa where the tested quinoa genotypes reached maturity stage earlier in the first season than the second season. Yearly variability of growth duration due to different weather conditions as found in this study has been highlighted in other experiments conducted in Italy^19^ and in Egypt^42,55^.

Our findings are consistent with those of^15^, which reported a non-significant correlation between the grain yield and the days to flowering and the harvest maturity, which suggests that these two parameters only slightly influenced the yield under open field conditions of hot dry lands. While our results are not in line with^47^, which reported that, the fast-maturing varieties were typically among the highest yielding. In our case, although the Santa Maria genotype has recorded the shortest harvest maturity, it recorded the highest TWG.

### Yield variables

The values of TGW obtained in this study (1.61–2.68 g) are comparable to those recorded by^41^ in Turkey (TGW = 2.12–2.61 g), but lower compared to the ranges reported by^37^ in Andean region (3.0–4.7 g)^15^, in South Asia (1.4–3.7 g), Pulvento et al.^40^ in Southern Europe (1.8–3.6 g) and Präger et al.^57^ in Germany (1.2–3.3 g). The lower TGW obtained for the tested quinoa genotypes compared to the above-mentioned previous studies may be due to the delay of sowing time. In fact, early sowing ensures a better filling of quinoa grains with nutrients compared to late sowing^24,58^. A low TGW may result of insufficient nitrogen fertilization at the time of reserve accumulation in the grain^43^. In fact, it is probably due to high nitrogen inputs at the critical plant growing phase that Shams^42^ obtained higher TGW (2.5–4.5 g) compared to our case, despite the similarity of the pedoclimatic conditions of the two field trials.

The highest mean values of TGW were recorded by Santa Maria (2.68 g), while the lowest values were obtained by Q18 (1.61 g). It appears that the Santa Maria genotype was more efficient in translocating of photosynthates from leaves and stems to the developing panicle then to the seeds compared to other genotypes under our conditions. In fact, the long growth period of this genotype was conducive for better grain filling and grain weight. In addition, the high TGW recorded with this genotype may be attributed to its relatively high resistance to diseases at the end-of-cycle which can be justified to its relatively high GSC compared to others genotypes (field observation). The observed genetic variability of quinoa TGW in this study has been also signaled in previous studies^31,57,59,60^.

The genotypic variability of yield in quinoa depend to the difference in response of different genotypes of this plant species to the combined effect of pedoclimatic conditions (temperature, rainfall, soil humidity, soil and water salinity), conditions of conduct of the field experience in each region (date and density of sowing, nature and level of fertilizers and irrigation) and the variation of quinoa genotypes used and their difference in residence to physiological diseases and crop pests. According to^52^, the yield potential of quinoa is the result of the effects of environmental factors and cultivation techniques together.

The grain yield recorded in this field trial remains low compared to the yields obtained in experiments carried under similar conditions (sowing in November and sandy soil) in Morocco^24^ and United Arab Emirates^21,22^, where GYd totaled 3.07 t/ha, 4.46 t/ha and 3.85 t/ha, respectively. The difference in yield between these experiments can be explained by the fact that the experiment of Morocco was irrigated with treated wastewater which is very rich in nutrients and organic matter. While, for the tests realized in Dubai, the plants were fertilized in addition to mineral fertilization with 40 t/ha of organic fertilizer and irrigated with low-salinity water (1–2.8 dS/m) which could explain the yield gap between these experiences compared to our case. The relatively low yield obtained in our case can also be related to the low temperatures at the time of flowering. Despite, the adaptation of quinoa outside the Andes, a low temperature during flowering stage can significantly reduce grain yield^47,48,61^. In fact, low temperatures measured in the month of January and February has probably affected quinoa development which might be a reason for not yielding high scores.

Moreover, while no major differences in terms of pedoclimatic conditions for both seasons (same environment), the differences in yields could only be attributed to differences in genetic structure of genotype tested in particularly to the variability of their response to the pedoclimatic conditions of the study region (soil texture, salinity of soil and irrigation water etc.). For example, high variability in salinity tolerance among quinoa genotypes has been reported^62,63^. Traditionally, only genotypes from the Bolivian Salares like (Santa Maria) were thought to have a high tolerance to salinity^64^. However, the results indicate clearly that the highest values of these traits were recorded with (Q102) genotype, while Giza1 genotype had the lowest values for both seasons. In fact, salinity tolerance in quinoa does not correlate with geographic distribution; varieties from coastal regions of Chile and high land areas outside the Salares ecoregion have similar or even higher salt tolerance levels^65^. According to^32^, a wild relative of quinoa (*Chenopodium hircinum*) was found to have a much higher salinity tolerance level than quinoa genotypes such as the case of Q102.Accordingly, these results reveal that quinoa Q102 proved success in hot desert regions with suitable grain yield (2.8 t/ha). It showed more drought tolerance as compared to the other genotypes tested in this trial.

Our finding of GYd for Q27, Q29 and Q18 are lower compared to those reported by^19^, that recorded GYd = 2.87, 2.75, 3.01 t/ha, respectively. However, our results are close to those of Bazile^10^ for Q18 (2.27 t/ha) and Q27 (2.17 t/ha). The genotypes which recorded high yields had also recorded the highest scores for morphological parameters. This was confirmed by the correlation analysis where the morphological characters showed a positive relationship with the GYd which was positive and highly significant for most characters (Fig. 6). Thus, the different morphological parameters seem to strongly and positively influence the GYd. In addition, our results are in agreement with^57^, which reported no negative correlation between TGW and GYd. However, they are in contrast to those of^66^ that reported a strong negative correlation between these two yield parameters.

### Grain quality (saponin and protein contents)

The variation of GSC in quinoa depends mainly on the genotype^16,42,67,68^. It is higher in bitter-flavor varieties than in sweet or low-saponin varieties. Quinoa contains saponins in the amount from 0.1 to 5%^69^. In general, the saponin levels (0.62–1.92 g/100 g DM) of the tested genotypes in this study can be regarded as high in comparison to previous studies reporting ranges of 0.48–1.14 g/100 g DM^70,71^, 0.10–1.80 g/100 g DM^31^ and 0.07–0.22 g/100 g DM^42^. The relatively high GSC levels in the tested genotypes can be related to the high salinity of soil and irrigation water because quinoa GSC increase under saline conditions^70,72^ and according to nitrogen addition^43,84^. According to^73^, salinity increase buildup of saponin in quinoa, whereas drought decreases this component^71^. Yet, the relatively high GSC obtained in this study are in agreement with some other studies^72,74^ reporting that a significant increase of saponins in arid conditions under irrigation as compared to humid conditions.

Regarding the comparison of GSC in quinoa genotypes tested, the genotype ‘Santa Maria’ showed significantly the highest concentrations (1.92 g/100 g DM). Indeed, saponin was reported to exert a strong insecticidal or protective activity against a broad range of insects, herbivores (e.g. birds) and even microbial infections^75,76^. This agrees with our field observations especially for Santa Maria genotype showing a low susceptibility to diseases and attack by insects and birds compared to others genotypes in particular Giza1with slightly higher GSC (field observation). In relation to its low organolyptic parameters (bitter taste) and the difficulty of its digestibility, saponin must be eliminated before consumption of quinoa^75^. This seems to be a favorable property of Giza1 due to its low content of this compound (0.62 g/100 g) compared to other genotypes tested (Fig. 5). According to^31^ genotypes Q18 and Q29 are sweet varieties (low-saponin contents < 1%) with average GSC of 0.57 g/100 g DM. However, in our case these genotypes recorded 0.78 and 0.87 g/100 g DM, respectively. By contrast to the same study that recorded a GSC of 1.51 g/100 g DM for Q27 genotype, considering it thus as bitter genotype, we recorded lower GSC of about 1 g/100 g DM for the same genotype. Also, by comparison to^11^, where the mean GSC in Giza1 had the highest value (0.42 g/100 g DM), in our case Giza1showed the lowest content (0.62 g/100 g DM). In the same trend, while Sajama genotype had the lowest value (0.18 g/100 g DM), in our case Sajama recorded an intermediate GSC (0.73 g/100 g DM). In this context, the differences observed in the varietal behavior of quinoa in the present study for GSC do not support the hypothesis that claims that this trait is largely determined by the genotype. The classification of quinoa genotypes according to their GSC as sweet or bitter genotype should not be generalized. In fact, while a genotype behaves as a sweet genotype in one region under specific agro-ecological conditions, it can change its behavior by manifesting as a bitter genotype in another region with different conditions. Quinoa GSC depends on the response of the plant to the interactive effect of genotype and agro-ecological conditions. These findings show that GSC depends on agro-environmental conditions and not only on genotype. On the other hand, GSC depends also on the growth stage of the crop, where it increases when passing from the branching stage to the flowering stage^15^. This justifies the significant positive correlation indicated by the current study between GSC and D-Fl (*p* < 0.001). As for, the positive and significant correlation observed between D-Mt and GSC, it can be explained by the increase in the synthesis of this compound by the plant in response to the approach of the summer period which is characterized by intensive attacks of this plant by different enemies of the crop.

The range of GPt (11.89–16.7%) obtained in this study were similar to those reported by^57^ (11.9–16.1 g/100 g DM). In comparison to other studies, the observed GPt levels are higher than those reported by^37^ (12.85–16.1 g/100 g DM), and^77^ (13.9–15.47 g/100 g DM)^78^, (9.15–15.53 g/100 g DM) and^42^ (11.03–13.77 g/100 g DM). However, Bhargava et al.^15^ and Stikic et al.^79^, indicated that GPt from various genotypes ranged between 12.55–21.02 g/100 g DM and 11–19 g/100 g DM, respectively; which were higher compared to our results. The variability in quinoa GPt between these studies may be related to the difference between ecological conditions and the genotype effect itself^6,57,78^. On the other hand, the high protein contents recorded under arid conditions of this study indicate that quinoa GPt was not strongly altered by the pedoclimatic conditions of the Sahara Desert in particularly the salinity of soil and water which confirm the results of^70^.

The total protein content was found within a range already described for quinoa grains (between 15 and 20 g/100 g DM)^74^ only for Q102, Santa Maria and Q27. While, Q18 and Giza1 recorded lower GPt of this range. In addition, grain proteins content in the present study was similar to that reported by^11^ for genotype Sajama (17.67 g/100 g DM), however, it was higher than that reported for Giza1 (15.5 g/100 g DM). The significant positive correlation (*p* < 0.001) between GPt and GYd does not confirmed the generally accepted idea according to which the increase in GPt occurs to the detriment of GYd^19,80^. However, Bhargava et al.^15^ indicated non-significant correlation between these two parameters.

## Conclusion

This study indicated that the achievement of high grain yield and good grain quality in quinoa cultivation is possible under the hot-arid extreme environmental conditions (high salinity and drought) of the Sahara Desert, provided that the cropping season occurs during November–April. Our finding helped to identify quinoa genotypes particularly suitable for cultivation under such conditions of this region. However, it should be noted that the conditions of the field trial allowed each genotype to express its potential separately. While, some genotypes are shown to be interesting in terms of yield, others have shown high performance in terms of quality (GPt and GSC). Consequently, the decision for growing specific quinoa genotype has to rely and respond to the production objective. Over the two cropping seasons, the seven genotypes tested showed no significant differences in the most agronomic performances, yield parameters (TGW and GYd) and grain quality traits (GPt and GSC), which indicates a strong potential of stabilization of these characters. Therefore, it should be possible to select better adapted genotypes with high yields and nutritional quality combined with salt-and drought-tolerance. Among all of the genotypes tested, it is possible to select Q102 followed by Q29 as the most promising parental genotypes for the development of high yields and quality genotypes, and for optimal adaptation to pedoclimatic conditions of hot drylands worldwide.

## Data Availability

The datasets used and/or analyzed during the current study are available from the corresponding author on reasonable request.

## Abbreviations

GLMM: Generalized linear mixed models
GPt: Grain proteins [g/100 g DM]
GSC: Grain saponin content [g/100 g DM]
GYd: Grain yield [t/ha]
D-Fl: Number of days from emergence to flowering
D-Mt: Number of days from emergence to maturity
NPn: Number of panicles per plant
PHt: Plant height [cm/plant]
TGW: Thousand grain weight [g]
